# Ventilation with the esophageal-tracheal Combitube during general anaesthesia: assessing complications in 540 patients

**DOI:** 10.48101/ujms.v128.9212

**Published:** 2023-04-21

**Authors:** Nicole Harrison, Sahra Pajenda, Lukasz Szarpak, Anna-Maria Buschsieweke, Mostafa Somri, Michael Frass, Bernhard Panning, Oliver Robak

**Affiliations:** a Department of Medicine I, Division of Infectious Diseases and Tropical Medicine, Medical University of Vienna, Vienna, Austria; b Department of Medicine III, Division of Nephrology, Medical University of Vienna, Vienna, Austria; c Department of Emergency Medicine, Medical University of Warsaw, Poland; d Department of Medicine I, Intensive Care Unit, Medical University of Vienna, Vienna, Austria; e Department of Anaesthesiology, Bnai Zion Medical Centre, Haifa, Israel; f Department of Anaesthesiology, Medical University of Hannover, Hannover, Germany

**Keywords:** Combitube, ETC, general anaesthesia, complications

## Abstract

**Background:**

The esophageal-tracheal Combitube (ETC) was developed for the management of difficult airways but can also be used for general anaesthesia.

**Methods:**

This clinical study collected data from patients undergoing anaesthesia with the ETC in order to assess the rate of complications.

**Results:**

Five hundred forty patients were ventilated with the ETC. In 94.8% (512/540), insertion was performed for the first time by the respective physician. The following minor complications were observed: 38.7% sore throat, 30.9% blood on tube as sign of mucosal lesions and 17.0% cyanotic tongue. Experience decreased the risk of mucosal lesions (odds ratio [OR]: 2.3, 95% confidence interval [CI]: 1.5–3.5). A higher than recommended volume of the oropharyngeal cuff was associated with blood on the ETC (OR: 1.5, 95% CI: 1.0–2.3) and tongue cyanosis (OR: 2.3, 95% CI: 1.4–3.7). Ventilation for more than 2 h was associated with tongue cyanosis (OR: 2.2, 95% CI: 1.6–3.1) and tongue protrusion (OR: 1.4, 95% CI: 1.1–1.9).

**Conclusion:**

We conclude that the Combitube may be used for short procedures requiring general anaesthesia, but the high rate of minor complications limits its value when other alternatives such as a laryngeal mask airway are available. The tested method appears safe regarding major complications, but minor complications are common. Adherence to recommended cuff volumes, experience with the ETC and limiting its use to surgeries lasting less than 2 h might reduce the rate of complications.

## Background

The Combitube (esophageal tracheal combitube, ETC; Medtronic, Dublin, Ireland) was developed for the management of difficult airway situations (1, 2). These are clinical situations in which a trained anaesthesiologist experiences difficulties with bag-mask ventilation, difficulties with endotracheal intubation (ETI) or both (3). However, its ease of usage makes the ETC applicable for utilisation in elective surgery, similar to the laryngeal mask airway (LMA) (4). In contrast to the LMA (5–8), there are only few studies for the use of the ETC during general anaesthesia (9, 10).

Moreover, several authors recommended the use of the ETC for elective surgery (9, 11–14). The advantages of the ETC include inserting and securing an airway without the need for neck or head positioning, minimised risk of aspiration, firm anchorage of the device in the oropharynx after inflation of the respective balloon, application of high respiratory pressures and the fact that ventilation works equally well both in the tracheal and the oesophageal position (2, 15, 16).

Several smaller studies already evaluated the use of the ETC during routine surgery with an overall of 524 patients (9, 10, 12, 17–20). Primary concerns are uncertainties about the complication rate of the device, the difficulty of insertion and the feasibility of utilisation during general anaesthesia. While only few major complications are described in the literature, Vezina et al. found that utilisation of the ETC during blind insertion into the airway of critically ill emergency patients resulted in a 4.3% incidence rate of major complications (emphysema, tracheal injury, oesophageal perforation and upper airway bleeding) (21). Similar complication rates are also found after conventional ETI (22). However, several confounding factors have to be considered when assessing the ETC in an emergency setting. The traumatic impact of chest compressions as well as trauma or illness leading to intubation can result in complications. Therefore, assessing the ETC as an airway device in the standardised situation of an elective surgical procedure allows for an elimination of these confounding factors and for an evaluation of complications directly associated with ETC. We therefore collected data from patients undergoing routine anaesthesia with the ETC in order to assess the rate of complications and whether the ETC can be used for mechanical ventilation in daily practice. Insertion of an ETC is not a routine strategy for airway management in elective surgery in our institute. The reason for the application of this device for elective surgery was that operators should be prepared for emergency situations.

## Methods

This clinical study was approved by the local Ethics Committee of the Medical University of Hannover, Germany (No. 3846/2005). Data were collected at the Department of Anaesthesiology at the Medical University of Hannover. This was a clinical study including patients with ETC used for airway management. Patients were given oral and written information and signed informed consent at least 2 days prior to intubation. Patients’ age, gender, body mass index (BMI) and type of planned surgeries were recorded.

The patients’ airways were classified according to Mallampati (23). Patients were monitored by routine non-invasive measures including electrocardiogram, pulse oxymetry, temperature, non-invasive blood pressure measurement and capnography. The patient’s mouth was opened with one hand, and the ETC was inserted parallel to the patient’s chest, so as to facilitate insertion until the black rings on the ETC were positioned at the level of the patient’s maxillary teeth. Depending on the preference of the respective physician, the ETC was either positioned blindly and thereby usually entering the oesophagus or by using a laryngoscope intentionally inserting it into the esophagus.

The ETC is available in two different sizes: 37 F SA (small adult) for patients up to 180 cm and 41 F for patients above 180 cm (24). However, in literature, the 37 F version was used more often because of its ease of insertion (9, 12, 17, 24–26), and the fact that it was successfully used up to a patient’s height of 198 cm (25, 27).

The volumes used to inflate the oropharyngeal and oesophago-tracheal cuffs of the 37 F and the 41 F were 85 and 100 mL, respectively, for the proximal balloon (28) and 10 mL for the distal balloon as recommended by the manufacturer. The oropharyngeal and esophago-tracheal cuffs were inflated successively one after another. Correct placement of the respective device was confirmed by auscultation, oxymetry and end-tidal CO_2_-concentration (EtCO_2_) measurements. In case of failure to ventilate sufficiently or place the ETC correctly, a conventional endotracheal airway was used as rescue device.

Block volumes of both cuffs were recorded.

With the ETC in place, tongue cyanosis and protrusion were recorded if observed. Tongue protrusion measurement by help of a measuring tape was part of the routine ETC use. After removal, the ETC was inspected for traces of blood. Patients were questioned about pain in the throat or oesophagus during postoperative rounds. 24 h later, patients were asked again with regard to potential discomfort. Minor complications were defined as lasting for less than 24 h, while major complications lasted for more than 24 h. Besides anesthesiologists, emergency physicians and specialists in internal medicine inserted the ETC.

### Statistical analysis

Data were tested for normal distribution, and analysis was performed by univariate logistic regression; a *P* < 0.05 was considered statistically significant. Results were presented as odds ratios (ORs) with 95% confidence intervals. Descriptive data were presented as mean with standard deviation (SD). Analysis was done with SPSS version 23 (IBM, Armonk, NY, USA).

## Results

### General characteristics of study population

Five hundred forty patients underwent general anaesthesia for an elective surgery and were ventilated with the ETC during the entire procedure without switching to another airway device. To ensure that the observed complications were associated with ETC, only these patients were included in the final analysis. The database contained a further 88 patients where the ETC was initially used but then switched to another device. The reason for switching was that surgery lasted for more than 2 h. As observations as well as complications in these patients could have been caused by both airways, those patients were excluded from analysis.

In 94.8% (512/540), insertions of the ETC were performed for the first time by the respective physician.

The characteristics of the study population (*n* = 540), the medical procedures and the characteristics of the ETC are summarised in Table 1.

**Table 1:** General characteristics of study population, medical procedures, and ETC.

Characteristics of study population (*n* = 540)	Mean ± SD or total number and percentage
**Age** (*n* = 532), years	56.9 ± 17
**Sex** (*n* = 540) Male Female	430 (79.6%) 110 (20.4%)
**Weight** (*n* = 502), kg	77.8 ± 17.2
**Size** (*n* = 502), cm	173.1 ± 9.4
**BMI** (*n* = 502)	25.8 ± 4.9
**ASA physical status** (*n* = 517) ASA I (healthy) ASA II (minor illness) ASA III (severe illness) ASA IV (life-threatening illness) ASA V (moribund)	5 (1%) 89 (17.2%) 400 (77.4%) 12 (2.3%) 11 (2.1%)
**Type of surgery or medical procedure** (*n* = 540) Pacemaker implantation Percutaneous ethanol injection Electroconvulsive therapy Magnetic resonance imaging Percutaneous transhepatic choledochal drainage Endoscopic retrograde cholangiopancreatography Other surgeries	200 (37.0%) 165 (30.6%) 50 (9.3%) 17 (3.1%) 15 (2.8%) 4 (0.7%) 80 (14.8%) 9 (1.7)
**Indication for ETC** (*n* = 530) Training Special indication* Anticipated difficult airway Difficult intubation Rapid-sequence induction	344 (64.9%) 63 (11.9%) 57 (10.8%) 50 (9.4%) 16 (3%)
**Qualification of physician** (*n* = 533) Anaesthesiologist Other physicians	396 (74.3%) 137 (25.7%)
**Experience with ETC** (*n* = 538) Beginner (1–3 times ETC use) Experienced (>3 times ETC use)	210 (39%) 328 (61%)
**ETC size** (*n* = 508) 37 F (SA) 41 F	459 (90.4%) 49 (9.6%)
**Insertion method** (*n* = 517) Without laryngoscope With laryngoscope	142 (27.5%) 375 (72.5%)
**Number of number of attempts** (*n* = 456) Successful at first trial** More than one trial necessary	418 (91.7%) 38 (8.3%)
**Duration of ETC in situ** (*n* = 471) <1 h 1 to 2 h >2 h	175 (37.2%) 191 (40.6%) 105 (22.3%)
**Block volume oropharyngeal cuff** (*n* = 452), mL 37 F (85 mL recommended) 41 F (100 mL recommended)	92.5 ± 19 103.8 ± 13
**Block volume esophago-tracheal cuff** (*n* = 441), mL 37 F (10 mL recommended) 41 F (10 mL recommended)	8.8 ± 2.1 11.9 ± 3.8

ASA: American Society of Anesthesiologists Physical Status Classification System; BMI: body mass index; ETC: esophageal-tracheal Combitube; SD: standard deviation.

* Special indication: teeth assessment, vocal cord protection, electroconvulsive therapy, MRI, avoiding movement of cervical spine;

** Successful ventilation assessed by lung auscultation.

### Observations and complications with ETC during general anaesthesia

Observations and minor complications were documented in 86% (464/540) of patients ventilated exclusively with ETC during general anaesthesia. The most frequent observation was protrusion of the tongue because of ETC in 50.4% of patients (272/540). A significant protrusion of more than one centimetre was observed in 10.64% (57/540). On average, the tongue protruded 0.57 (SD ±0.63) centimetres during the procedure. The most often observed complication was sore throat occurring in 38.7% (209/540) of patients after use of ETC with 5.2% (28/540) of them complaining about severe pain. After removal of ETC, in 30.9% (167/540), blood was detected on the tube. In 24.1% of patients (130/540), only traces of blood were noted; in 5.2% (28/540), a moderate amount and in 1.7% (9/540), a significant amount of blood was found on the tube. A cyanotic tongue was observed in 17.0% (92/540) of patients. Only one case was reported where severe tongue cyanosis prompted a change to a different airway device. All complications disappeared within 24 h. No major complications or severe injuries (e.g. arterial bleeding, stridor, fractures, etc.) were noted in any of the patients ventilated with ETC.

### Risk factors for complications with ETC

Using univariate logistic regression, risk factors were calculated for the occurrence of the following complications: tongue cyanosis, protrusion of tongue, blood on the tube and sore throat (see Table 2). Size and weight of the patient were significantly associated with the risk for tongue cyanosis (OR: 0.96 and 0.98, respectively) or protrusion (OR: 0.95 and 0.98, respectively), but not the BMI. Female patients showed a higher risk for protrusion of tongue (OR: 2.4) and for suffering from sore throat (OR: 1.9). An American Society of Anesthesiologists Physical Status Classification System (ASA) of III or higher, meaning that the patient was severely ill was significantly associated with an increased risk for blood on the tube (OR: 2.0). Experience with the use of ETC (more than three prior intubations using this device) was associated with a decreased risk for mucosal lesions (OR: 1.6). The use of ETC by a physician other than an anaesthesiologist was associated with an increased risk for blood on the tube (OR: 2.3). A longer duration of the ETC in situ showed an increase for tongue cyanosis (OR: 2.2) and for tongue protrusion (OR: 1.4). A block volume of the oropharyngeal cuff that exceeded the recommended volume was associated with an increase of tongue cyanosis (OR: 2.3) and of blood on the tube (OR: 1.5). On the other side, a higher block volume for the esophago-tracheal cuff (more than 10 mL) was associated with a decreased risk for tongue cyanosis (OR: 0.5) and tongue protrusion (OR: 0.5).

**Table 2:** Risk factors for complications with ETC.

Risk factor for complication	Tongue cyanosis			Protrusion of tongue			Blood on tube			Sore throat		
	Total *n*	OR	95% CI	Total *n*	OR	95% CI	Total *n*	OR	95% CI	Total *n*	OR	95% CI
Age	428	1.0	0.99–1.0	421	1.0	0.99–1.0	433	1.0	1.0–1.03	384	1.0	0.99–1.0
Sex (female)	431	1.5	0.9–2.6	425	**2.4**	**1.4–4.2**	434	1.1	0.7–1.7	385	**1.9**	**1.1–3.1**
Size	425	**0.96**	**0.94–0.99**	418	**0.95**	**0.93–0.98**	425	1.0	0.98–1.0	377	1.0	0.98–1.0
Weight	425	**0.98**	**0.96–0.99**	418	**0.98**	**0.97–0.99**	425	1.0	0.99–1.0	377	1.0	0.98–1.0
BMI	425	0.95	0.9–1.0	418	0.97	0.93–1.0	425	1.0	0.96–1.1	377	0.97	0.93–1.0
ASA	417	1.5	0.8–2.9	411	0.7	0.4–1.2	418	**2.0**	**1.1–3.5**	372	1.2	0.7–2.1
ETC size	403	1.6	0.6–4.0	397	0.7	0.3–1.5	407	0.6	0.2–1.5	361	0.6	0.3–1.5
Insertion method	423	1.1	0.7–1.9	417	1.1	0.7–1.7	426	1.0	0.6–1.5	377	1.0	0.6–1.6
Number of insertion attempts	384	0.9	0.4–2.4	378	1.1	0.5–2.5	382	1.1	0.5–2.5	340	1.5	0.7–3.4
Qualification of physician	430	0.7	0.4–1.2	424	0.8	0.5–1.2	433	**2.3**	**1.5–3.5**	383	1.2	0.8–1.9
Experience with ETC	431	0.9	0.5–1.4	425	0.9	0.6–1.3	434	**1.6**	**1.1–2.3**	385	1.0	0.7–1.5
Duration of ETC in situ	412	**2.2**	**1.6–3.1**	405	**1.4**	**1.1–1.9**	412	0.9	0.7–1.2	361	1.2	0.9–1.6
Block volume oropharyngeal cuff	419	**2.3**	**1.4–3.7**	410	1.1	0.7–1.7	420	**1.5**	**1.0–2.3**	364	1.3	0.9–2.1
Block volume esoph.-trach. Cuff	414	**0.5**	**0.2–0.9**	406	**0.5**	**0.3–0.8**	416	0.9	0.6–1.4	356	0.6	0.3–1.0

ASA: American Society of Anesthesiologists Physical Status Classification System; BMI: body mass index; CI: confidence interval; ETC: esophageal-tracheal Combitube; OR: odds ratio; SD: standard deviation.

ASA: I-II (healthy or minor illness) versus III-V (very ill to moribund); ETC size: 37F versus 41F; Insertion method: with versus without laryngoscope; number of number of attempts: one attempt versus more than one attempt; Qualification: anaesthesiologist versus other physicians; Experience: Beginner (1–3) versus Experienced (>3); Duration of ETC in situ: <1 h, 1–2 h, >2 h; Block volume oropharyngeal cuff: recommended volume versus more than recommended (>85 mL for 37F, >100 mL for 41F); Block volume esophago-tracheal cuff: recommended volume versus more than recommended (>10 mL).

Bold values = Statistically significant data.

### Reasons for switching ETC to other airway device

The database contained 88 cases where the ETC was initially used for airway management but then changed to another airway device. ETC was changed for logistic reasons (e.g. long duration of surgery, *n* = 24) or was just intended for bridging before a tracheotomy or placement of a permanent airway device (*n* = 8). In 46 cases out of the 88 mentioned above, ETC had to be changed to another airway device because of problems with the ETC (reason unknown, *n* = 10). The following problems were noted: leakage of the ETC despite increase of the cuff volume (*n* = 16), insufficient ventilation (*n* = 16), high inspiratory ventilatory pressure (*n* = 10), hiatus hernia (*n* = 1), minor injury of the soft palate (*n* = 1), severe gastric reflux (*n* = 1) and severe tongue cyanosis (*n* = 1).

Considering the characteristics of these 46 patients, there were no major differences concerning age, sex, weight or size compared to patients with successful use of ETC (data not shown). However, the majority of patients was classified as ASA physical status III (95.7%, *n* = 44). Most elective procedures in this group were pacemaker implantations (65.1%, *n* = 28) or percutaneous ethanol injections of malignancies (27.9%, *n* = 12). The experience of the physician, the experience with ETC or the insertion method did not have a significant impact on having to change ETC to another airway device.

## Discussion

Overall, this study proved that ETC is a safe device for ventilation during general anaesthesia in minor elective procedures. Although minor complications like protrusion of the tongue or sore throat occurred frequently, major complications like symptoms lasting for more than 24 h or even rupture of the oesophagus, as reported previously (22, 29), had not been observed. The success rate for ventilation with ETC was about 92% taking into account that a change to another airway device was necessary in 46 cases (46/540). Furthermore, one should keep in mind that in 94.8% (512/540), insertion of the ETC was performed for the first time by the respective physician.

This success rate was slightly lower than reported by other studies, where ventilation was achieved in 97% (9) or even 99% (17) of patients with ETC. Major reasons for not maintaining ventilation with the ETC were leakage despite increase of the cuff volume (*n* = 16), insufficient ventilation (*n* = 10) or high inspiratory ventilation pressure (*n* = 10).

Protrusion or cyanosis of the tongue was observed in 50.4 and 17.0% of patients, respectively. The pressure exerted by the oropharyngeal cuff might impair venous blood flow from the tongue leading to cyanosis. In line with this, in the present study, we found that a higher than recommended cuff volume was associated with a higher risk for tongue cyanosis. Interestingly, a higher than recommended volume (>10 mL) of the oesophago-tracheal cuff reduced the risk for tongue cyanosis significantly. We do not have a verisimilar explanation for this observation. Another important factor influencing tongue cyanosis and protrusion was the duration the ETC was left in situ. The ETC is recommended for procedures lasting up to 8 h (30). A longer duration necessitates a change of the ETC to another airway device, especially because tracheal secretion cannot be removed by suction if placed in the oesophageal position. Theoretically, compression and cyanosis of the tongue could theoretically lead to postoperative oedema and subsequent airway compromise. However, out of 540 patients, only one case of severe tongue cyanosis was observed that led to removal of the ETC.

The most common minor complication described by other studies is superficial mucosal lesions (17, 19, 31). In previous studies, the incidence of traces of blood on the ETC after removal ranged from 10% (12) to 47% (9, 10, 32). Compared to the LMA or ETI, the ETC causes slightly more lesions (19). Most plausible reasons for this comprise laceration during insertion of the ETC, over-inflation of the pharyngeal cuff causing mucosal lesions and rigidity and anterior flexion of the ETC during insertion (Lipp manoeuvre) (21, 33–35).

Most comparisons to LMA were done in the prehospital setting. In a series of 470 patients reported by Rumball and colleagues (36), the ETC was rated best when compared with the pharyngeal tracheal lumen airway (PTLA), the LMA and bag-valve ventilation with mask and oral airway. The study was performed by emergency medical technicians (EMTs) in patients with cardiorespiratory arrest. Successful insertion rate was significantly higher (*P* < 0.01) with the ETC (86% vs. 73% LMA) despite the fact that some of the EMTs had been previously trained to use the LMA in the operating room. Mean PaO_2_ and mean exhaled volume were highest with ETC confirming previous studies. No aspiration with the ETC was noted in autopsies (36). Tanigawa et al. have studied emergency intubation of 12,020 cases of prehospital cardiac arrest (31). Successful insertion rates on the first attempt were 82.7% with the oesophageal gastric tube airway (EGTA), 82.4% with the ETC and 72.5% with the LMA (*P* < 0.0001). The rate of failed insertions was 8.2% with the EGTA; 6.9% with the ETC and 10.5% with the LMA (*P* < 0.0001). Successful ventilation could be achieved in 71% with the EGTA; in 78.9% with the ETC and 71.5% with the LMA (*P* < 0.0004). Six cases of aspiration were reported in the LMA group, whereas nine cases of soft-tissue injuries, including oesophageal perforation, were reported in the ETC group (31). The authors conclude that the ETC appears to be the most appropriate choice among the airway devices examined.

In this study, we noted blood on the tube as a sign for mucosal trauma in 30.9% of patients. This is a higher rate than reported by other studies (9, 12) although a laryngoscope was used for insertion in the majority of cases (72.5%). The qualification or the experience of the physician conducting the intubation decreased the risk of mucosal lesions. Experience with the ETC of at least three prior intubations was associated with a decreased risk for blood on the ETC. An increased volume of the oropharyngeal cuff was associated with a higher risk for blood on the ETC as a higher volume can cause more injury to the mucosa. Patients with an ASA physical status of III or higher had also an increased risk for bleeding as a result of mucosal lesions. Only one case was observed with an injury of the soft palate prompting removal of the ETC.

Interestingly, women were at higher risk for tongue protrusion and for sore throat after use of ETC. This might be related to the female anatomy of the pharynx; however, we do not have sufficient data to support this claim. Complications might often occur because of the individual anatomy of the patient, which was not evaluated in this study. Overall, sore throat occurred in 54.3% of patients and was therefore the second most common complication associated with the ETC.

A major strength of this study is the large cohort of patients. During the primary analysis, only patients who were ventilated with the ETC during the entire procedure were evaluated in order to ensure that all observed complications were directly associated with the ETC. A total of 540 patients were included, which is a considerably higher sample size than in other studies (9, 10, 12, 17, 20).

Another advantage is the controlled setting of an elective surgery with standardised procedures compared to an emergency setting with several confounding factors. Many complications described in such studies (21) can be attributed to the associated risks of cardio-pulmonary resuscitation including chest compressions or trauma.

A further outstanding difference of this study as compared to other studies about ETC in elective surgery is the heterogeneity of the study population as well as the variety of elective procedures where ETC was used for ventilation. In contrast to other studies, the majority of patients (82%) suffered from severe illnesses and co-morbidities (ASA physical status III or higher). This might partly explain the slightly lower success rate as well as the higher rate of minor complications.

As a possible limitation, it can be argued that a true prospective study with a more strict protocol would have been ideal. However, this study deals with complications primarily. In this way, it appears even more objective since there was no danger of bias at the time of documentation in regard to correctly reporting complications, as the investigators had not been involved in the process of airway management. Furthermore, there are several prospective studies published so far with the use of the ETC in elective cases.

Overall, the evaluation of ETC in general anaesthesia is limited by the fact, that ETC was developed primarily for use in emergency situations. Of course, emergency situations are associated with different complications than elective surgeries; therefore, this study can only evaluate the ETC in this specific context.

Considering this large cohort of patients including severely ill persons, this study showed that the ETC is a safe airway device for a broad range of minor elective surgeries reporting no major complications and a 92% success rate for maintaining ventilation during the entire procedure. We conclude that the Combitube may be used for short procedures requiring general anaesthesia but the high rate of minor complications limits its value when other alternatives such as a LMA are available. The tested method appears safe regarding major complications but minor complications are common. Adherence to recommended cuff volumes, experience with the ETC and limiting its use to surgeries lasting less than 2 h might reduce the rate of complications.

## Disclosure statement

Michael Frass has received royalties for the invention of the Combitube.

## Notes on contributors

OR, BP, NH, SP, LS and MF designed the study and drafted the manuscript. OR, MF and BP developed the theory and performed the statistical analyses. MS, SP, NH and OR verified the analytical methods. MF and LS supervised the findings of this work. All authors discussed the results and contributed to the final manuscript.

## ORCID

Nicole Harrison http://orcid.org/0000-0001-5971-0662

Lukasz Szarpak http://orcid.org/0000-0002-0973-5455

Mostafa Somri http://orcid.org/0000-0002-3814-1402

Michael Frass http://orcid.org/0000-0003-4140-4258

Oliver Robak http://orcid.org/0000-0002-3238-194X
